# Symptoms at stroke onset as described by patients: a qualitative study

**DOI:** 10.1186/s12883-024-03658-4

**Published:** 2024-05-03

**Authors:** Jenny Andersson, Åsa Rejnö, Sofie Jakobsson, Per-Olof Hansson, Susanne J Nielsen, Lena Björck

**Affiliations:** 1 Department of Molecular and Clinical Medicine, Sahlgrenska Academy, University of Gothenburg, Gothenburg and Sahlgrenska University Hospital/Östra Hospital, Diagnosvägen 11, Gothenburg, 41650 Sweden; 2 Region Västra Götaland, Department of Medicine Geriatrics and Emergency Medicine/Östra, Sahlgrenska University Hospital, Gothenburg, Sweden; 3 Stroke unit, Department of Medicine, Skaraborg Hospital, Skövde, Sweden; 4 Department of Health Sciences, University West, Trollhättan, Sweden; 5 Skaraborg institute for Research and Development, Skövde, Sweden; 6 Institute of Health and Care Sciences, Sahlgrenska Academy, University of Gothenburg, Gothenburg, Sweden; 7 Department of Cardiothoracic Surgery, Sahlgrenska University Hospital, Gothenburg, Sweden

**Keywords:** Patients, Stroke, Symptoms, Interviews, Content analysis

## Abstract

**Background:**

Stroke is a common and severe disease that requires prompt care. Symptom expressions as one-sided weakness and speech difficulties are common and included in public stroke campaigns. For some patients stroke can present with subtle and less common symptoms, difficult to interpret. The symptom severity assessed by the National Institutes of Health Stroke Scale has decreased, and symptoms at onset may have changed. Therefore, we aimed to investigate how patients describe their symptoms at the onset of a first-time stroke.

**Methods:**

The study used a qualitative descriptive design and conventional content analysis. Data were collected through recorded interviews with 27 patients aged 18 years and older hospitalised with a first-time stroke between October 2018 and April 2020. Data were analysed on a manifest level.

**Results:**

Symptoms at stroke onset were presented in two themes: *Altered Reality and Discomfort* and *Changed Body Functions* and described in five categories. Various types of symptoms were found. All symptoms were perceived as sudden, persistent, and never experienced before and this appear as a “red thread” in the result. Regardless of symptom expressions, no specific symptom was described as more severe than another.

**Conclusions:**

Stroke symptoms were described with a variety of expressions. Many described complex symptoms not typical of stroke, which can make it difficult to recognise the symptoms as a stroke and delay medical care. Public stroke campaigns should emphasize the importance of seeking medical care at the slightest suspicion of stroke and could be designed to help achieve this.

**Supplementary Information:**

The online version contains supplementary material available at 10.1186/s12883-024-03658-4.

## Background

Stroke is a common cause of mortality and morbidity worldwide [1] and associated with devastating consequences [2]. Rapid treatment in ischemic stroke with reperfusion treatment is essential to minimizing brain damage [1], and reducing the time from symptom onset to medical care is often imperative for a favourable prognosis [3, 4]. However, the time from symptom onset to medical care remains long for many patients [5, 6]. Patients’ inability to interpret stroke symptoms accurately has proven to be troublesome [7, 8], especially when symptoms are perceived as less serious or puzzling [9]. Even for healthcare providers, it can be difficult to interpret symptoms as a stroke [10]. The diversity of symptoms at stroke onset could lead to misinterpretations and cause delay [11, 12].

Stroke is a complex disease with several different expressions of symptoms [13]. Public campaigns have been developed, both nationally and internationally, to increase knowledge of stroke symptoms and the importance of seeking emergency medical care when experiencing or suspecting stroke symptoms [14, 15]. Common symptoms such as sudden weakness in limbs, face drooping, paralysis or slurred speech [16, 17] are included in stroke campaigns [18, 19]. Yet, some patients may present with less typical stroke symptoms, such as dizziness, vision problems or cognitive impairment [20–22].

As assessed by the National Institutes of Health Stroke Scale (NIHSS) [23], stroke severity scores have declined over the last two decades, both at emergent visit and discharge [24] regardless of sex and age, in all stroke types [24, 25]. It is consequently reasonable to assume that symptoms at stroke onset may have changed from what has commonly been considered typical symptoms. Previous studies have explored stroke symptoms expressions [26] in relation to campaigns [27] and decision making on seeking care [28]. To our knowledge, no studies have recently explored how patients describe their stroke symptoms at onset. Based on this there is a gap in the literature. Therefore, we aimed to investigate how patients describe their symptoms at the onset of a first-time stroke.

## Methods

This interview study used a qualitative descriptive design and conventional content analysis was utilised [29]. The Standards for Reporting Qualitative Research checklist was used (Additional File 1) [30].

### Setting and patients

We included 27 patients aged 18 years and older, hospitalised with a first-time stroke during October 2018 to April 2020, at 3 stroke units in Västra Götaland Region, Sweden, 2 at a university hospital and 1 at a local hospital. This study was part of a larger project [31]. Inclusion criteria was having a first-time stroke and being able to participate and interviewed within 4 weeks of symptom onset, and before hospital discharge. Stroke were defined according to the International Statistical Classification of Diseases, 10th Revision as I61, I63 or I64. No distinction was made between ischemic or haemorrhagic stroke. Exclusion criteria were transient ischemic attacks and subarachnoid haemorrhage, inability to make independent decisions, inability to express or understand the Swedish language, or too severely affected to participate. All participants received both oral and written information about the purpose and design of the study before inclusion. Information was provided about voluntary participation and terminating participation at any time without consequences. All included patients gave oral and written consent. Neither the interviewer nor any of the authors were involved in the patients care or treatment. The study was approved by the Regional Ethics Review Board in Gothenburg (Dnr: 487 − 18). The study complies with the principles of the Declaration of Helsinki.

### Data collection

The patients were identified by screening medical records (JA). A purposeful sampling strategy was used to achieve a variety in age and sex. All data on symptom onset was collected from patient interviews. The patients were invited to participate in the study by staff at respective units that not was involved in the study. Each patient was interviewed by JA at one single occasion, the majority within 2 weeks of stroke onset and before hospital discharge. The first author performed semi-structured in-depth interviews using an interview guide (Table 1). The interviews were recorded and transcribed verbatim to be analysed (JA). Each interview began with the open-ended question, “Can you please tell me what happened when you experienced the stroke symptoms?” No prepared ‘yes or no’ questions were used. Data collection ended when data was considered to be of enough depth and variation to answer the aim. The median duration of the interviews was 28 min (range 13–56 min). The interviews were supplemented with questions about demography presented in Table 2.

**Table 1:** Interview guide

Interview guide: Symptoms at stroke onset as described by patients
**Introduction:** Briefly describe what the interview study is about State the purpose of the study: • To investigate how patients describe their symptoms at the onset of a first-time stroke
**Main questions:** • Can you tell me what it was like when you first had your stroke? • Can you describe what symptoms you experienced? • Can you describe those symptoms? • How did you experience those symptoms?
**Closing question** • Is there anything else you would like to add?

**Table 2:** Baseline characteristics in patients with a first-time stroke

	Total *n* = 27	Men *n* = 16	Women *n* = 11
Age range, years	34–92	34–92	38–90
Median age, years	70.4	67.8	73
Mean age, years (standard deviation)	67.9 (15.6)	67.5 (14.1)	68.2 (18.6)
Atrial fibrillation, n (%)	1 (3.7)		1 (9.1)
Ischemic heart disease, n (%)	1 (3.7)		1 (9.1)
Hypertension n, (%)	17 (63)	11 (68.8)	6 (54.6)
Diabetes, n (%)	6 (22.2)	5 (31.3)	1 (9.1)

### Data analysis

The content analysis was inductive to capture and reproduce verbatim the patient’s description of symptoms at stroke onset. Since the data analysis not focused on the underlying meaning associated with the symptom experience, themes were constructed based on the content expressed in the interviews. A schematic overview of the analysis process is given in Table 3. After transcription of the recorded interviews, the analysis began with all interviews being read through as a whole to get an idea of the content. The meaning units were sorted out by JA, guided by the aim of the study. The meaning units were then condensed and given codes, labels that captured the content of the meaning unit, by JA and ÅR. The codes were grouped according to differences and similarities and arranged in preliminary categories representing the manifest content by JA, ÅR, SJ and LB. The preliminary categories included symptoms of the same type of character. The categories were then gathered based on symptoms of similar character. The themes and categories were tested on the data as whole and modified by JA, ÅR and LB to ensure the representation of the symptoms described. Finally, two themes and five categories were found to represent the result. The first-time experience appeared as a “red thread” binding the themes together. The final categorization was discussed until consensus was reached and agreed upon by all the authors. All shown quotations were translated from Swedish to English.

## Results

The study comprised 27 patients: 16 men (median age 67.8) and 11 women (median age 73) of which 37% were living alone (Table 3). The result is presented in two themes: *Altered Reality and Discomfort* and *Changed Body Functions* with five categories reflecting how patients describe their symptoms at onset when experiencing a first-time stroke (Table 4).

**Table 3:** Analysis process

Interview text	Meaning unit	Condensation	Code	Category	Theme
I felt off if you say so. I didn’t feel that I was well and perky but… but that it was something. (P5)	Felt off if you say, not feeling well and perky, it was something.	Felt off, not feeling well and perky, it was something.	Undefined feeling as off, not well, not perky	*Experience of illness*	*Altered Reality and Discomfort*
Then I would go out into the kitchen and pass by a small chest of drawers that I have there: my dear! I think I said that to it too: “move so I can get to the kitchen”. (P23)	Go into the kitchen, passed a small chest of drawers: my dear. Said that to it too: “move so I can get to the kitchen”.	Going to the kitchen, passing a drawer that seems to be getting in the way, it asks to move.	Experiencing furniture suddenly moves	*Changed perception of reality*	
Especially the slurring, I think I noticed it first. That it became really hard to talk. (P7)	The slurring, I noticed first. Became really hard to talk.	Noticing slurred speech, hard to talk.	Slurred speech, strained talk	*Loss of bodily functions*	*Changed Body Functions*
Then something just happened… I just slid down. Slowly, slowly to the floor. (P18)	Something just happened… I just slid down, slowly, slowly to the floor.	Something just happened, slowly sliding down to the floor.	Suddenly sliding down	*Loss of bodily control*	

**Table 4:** Overview of themes and categories

Themes	Categories
Altered Reality and Discomfort	Sensation of illness
	Experiences of discomfort
	Changed perception of reality
Changed Body Functions	Loss of bodily functions
	Loss of bodily control

During the initial onset of stroke, symptoms varied among the patients. Regardless of symptom, none of the patients had ever experienced anything similar. Never experienced variety of distressful and not understandable symptoms appeared to be the “red thread”. Symptoms were described as evident and sudden even when the symptoms were insidious or subtle. The symptoms were described as persistent and always present regardless of whether they occurred simultaneously or more symptoms were added subsequently. No symptom was described as more severe than the other. Several descriptions were given of both typical and unusual symptoms of varying character.

### Altered reality and discomfort

A diffuse, undefined ***Sensation of illness*** never experienced before was triggered during the onset of stroke. Feeling ill and tired were described. The perception of being ill differed from previous experiences of illness and was considered as “weird.” The experience was “different somewhat” and “hard to explain.” Previous experiences of not feeling well did not match the event. A strange feeling in the body, without being able to define it more closely, an unpleasant feeling “that something wasn’t right came on”. An unexplained, overwhelming tiredness that was exhausting and devastating occurred, and it was “impossible to carry on as usual”. An urgent need to sleep,” not feeling energetic” or an undefinable feeling of becoming ill were described.

*´I woke up not feeling well, but I don’t know what. A little tired…getting sick. I still wore the dressing gown all day…I don’t think I’ve been in my bed during the day for over 50 years*. ´ (P23).

Experience of discomfort of various kinds and intensities were reported. Discomfort could for example be described as dizziness which was lurking or striking. The dizziness was experienced as “unpleasant” or “awful” but always obvious even when it was fluctuating. The dizziness was never experienced before even though many tried to compare it to previous experiences of dizziness. Some were completely overcome by their giddiness and had an urge to shout their discomfort outright: “It’s terrible, it’s terrible, help me.” The dizziness affected the ability to move around. Some described feeling nauseous and vomited due to the dizziness. A discomfort as being drugged or drunk or even a stronger sensation, “heavily intoxicated” occurred. Some compared the discomfort to “a hangover” with sporadic or escalating nausea.

*´I said,” it´s spinning, the whole world is spinning”. It was horrible. I completely lost control. I lost it and said, “You must call Dad; he must come home. Say I´m dizzy”. ´* (P20).

A discomfort as feeling strangely warm or feeling hot inside the head or neck occurred as different types of headaches. The headaches were described as rushing, insidious or sudden headaches of a “never-before-experienced” nature. The headaches were different from earlier experiences; for some “intense”, for some “widespread”, and for some like a “needle”. The headache could sit like “a single point”. For others, the headaches were “crawling,” “grinding”, and “mashing” or “flashing” sensations. Others had a creeping headache that subsided or came and went in intensity. For some, “it felt like something heavy was lying on my head”, feeling like a massive pressure but for others as a stabbing pain that came in intervals.

*´I can say that it was because it was on the right side and it… what can I say… it stung in some strange way. So, it wasn’t like it was just sitting around grinding all the time. ´* (P25).

Patients related an emerging ***Changed perception of reality*** and altered self-perception. Descriptions of visual illusions such as objects moving around “cupboards moving and getting in the way”. Walls, paintings, and windows fell, and cabinets overturned.

*´ And then suddenly, when I turned from the sink, just… the refrigerator started to move. Oh… So, I quickly oh not to support the fridge but to support myself huh´.* (P2)

Visions of objects that did not exist such as beautiful paintings and human figures walking around were described. They also included flashing lights, double vision, and sound distortions. An altered reality with a feeling of unreality set in, described as being in a “science fiction movie”. Feelings of their surroundings moving “in slow motion” occurred.

Some described an altered self-perception as perceiving oneself as normal to becoming inexplicably changed. A feeling of being “dazed” by a new character. Some described it as being in and out of consciousness with blackouts, others like being “awake but not knowing anything”. Some perceived themselves as “between wakefulness and sleep” with only fragments of memory. Becoming a different person and no longer able to orientate at home, feeling giddy and perceiving oneself as confused. There were descriptions of strange thoughts and no longer perceiving oneself as oriented or “rational anymore”.

*´I recognized the paintings on the wall as mine, but I didn’t think I was home, so I was so surprised at what they were doing there´.* (P26)

Some who exhibited confusion and did not behave as usual or had affected speech were unaware of this, saying it was noticed by others around them. Others appeared unconcerned about the symptoms despite perceiving them and paid no attention to them. Others did not recognize parts of their body, such as a leg, as theirs.

### Changed body functions

Vivid descriptions of ***Loss of bodily functions*** were part of the narratives of symptoms. Arms and legs were described as “no longer working” and “could not be managed”.

*´Then my legs became like spaghetti. So, I said, “No, now something is not right”. I said, “I can’t walk”.* (P9)

“Slurred speech”, with speech problems that worsened progressively or difficulty “getting the words out right” were described. Speaking as “being drunk” of alcohol. A hanging corner of the mouth – their “face was crooked” – was also described. Also, visual impairment with difficulties seeing details or reading text occurred.

*´Then I also thought that this vision thing, that it began to shift, began to disappear, or fade away. And most of all, I found that when I looked at faces on TV, I couldn’t grasp what I was seeing. ´* (P10).

A negative impact on the senses contributed to impaired functions. Some described bodily sensations as “tingling”, “numbness”, “weakness”, or “dullness” in the body or a body part. Sensations “like electricity in my arm” or “no longer able to feel my feet”. Reduced sensation of hands or feet made it impossible to use the limb. Some described difficulties in completing tasks, such as dressing or buttoning, due to clumsy fingers, being unable to grip or reduced strength in the hand. Some ate with only one piece of cutlery or managed to drive a car even though only one leg worked. Their “arm was disloyal and no longer obeyed”; it was described as “crooked” and “unmanageable”.

Descriptions of ***Loss of bodily control*** suddenly developed. Some felt unsteady reducing the control over the body. Others fell on the floor or collapsed inexplicably. The whole body “had stopped working” and could not be controlled anymore. Experiencing new symptoms that began “out of the blue” and “so fast that no one knows what is going on”.

*´There was nothing! It came like a bolt from a clear sky. It’s called a stroke. And that was that. ´* (P18).

A weakness affecting the ability to control the body was told about. A need of help from others since it was impossible to stand up and walk or to sit up, resulting in “falling out of bed” and “knocking things over”. Feeling unsteady and feelings of paralysis resulted in loss of control being unable to move: “you lost your step”. Others tripped or “slid to the floor” without the ability to prevent it. Poor balance was attributed to “spinning and rocking a lot” making it hard to stand up. Many reported an imbalance that made it impossible to continue as usual. A need for support to move, “holding on to furniture” was described.

*´ Because it was balance problems that made me aware that something was not quite right. Because I usually don’t need a wall to support me, at all. ´* (P7).

For some, half of the body was “completely exhausted” and felt sluggish. Patients related that they staggered and had coordination difficulties. The body “started to hang” and was described as “completely gone” and “feeble”. It was hard and a strain to move. The body was described as a “lump of jelly”, weak, flimsy, and limp.

## Discussion

This study shows the complexity and diversity of symptoms at stroke onset. The patients’ experiences of the symptoms were described as new and differed from earlier experiences and appeared as a “red thread” through the descriptions. The results are presented in two themes; *Altered Reality and Discomfort* and *Changed Body Functions*, with categories that reflect how the patients describe their symptoms at stroke onset. The symptoms were persistent, obvious and could appear simultaneously. None of the symptoms were perceived as more severe compared to others. Patients were distressed regardless of symptom expression.

Typical stroke symptoms are often described as sudden numbness or weakness in the face or body [16, 17]. But several of the patients in this study described diffuse or insidious, less typical symptoms [21, 22]. Although, all patients stated that the symptoms were perceived as a new experience regardless of symptom expressions. It is known that varying symptoms could be due to different causes of stroke as well as the location of the brain damage [20] Even though this study did not collect data on location and spread of brain damage these factors may explain the richness of descriptions of less typical symptoms. Descriptions of initial feelings of illness without obvious symptoms were described. Other symptoms described were feelings of altered reality that affected the perception of one’s surroundings or a clouded mind. Many symptoms were described as difficult to explain, not tangible, and with different intensities. Difficulties with patients interpretations of subtle symptoms have also been reported in previous studies [26, 32]. Symptoms not perceived as severe, sudden, or devastating may not be recognized as stroke, neither by the patient nor by health care providers [33, 34]. Varying symptom descriptions could lead to misinterpretation of subtle stroke symptoms and have been shown to lead to a delay in healthcare contact [5, 35]. The degree of severity is not decisive for identifying the symptom as stroke [36] while the degree of severity can be crucial for seeking medical care [34]. Hence, difficulties interpreting minor stroke symptoms could be a barrier [35].

More effective treatment of risk factors such as high blood pressure, atrial fibrillation and diabetes has contributed to a decreased mortality rate in stroke and to less severe and disabling strokes [1, 37]. This change in severity could have reduced the symptoms at stroke onset [38, 39]. This is in line with our findings which indicate that symptoms at stroke onset may have changed and vary from what previously have been described as typical stroke symptoms [20]. Still, stroke remains a very serious disease [1] that requires prompt assessment to reduce brain tissue damage [39]. Several patients in this study may have been at risk for delayed care as many perceived symptoms as subtle and manageable. So, correctly interpreting even minor symptoms of stroke as serious is of the utmost importance [40]. As found in this study relatives or others may be the first noticing symptoms which may lead to faster medical care [28].

Descriptions of stroke symptoms by the patients might differ from those in the stroke awareness campaigns [18, 41, 42] which is partly in line with our findings. It is known that stroke campaigns focus mostly on the most common stroke symptoms [19, 43] which can lead to rarer symptoms being misunderstood. The patients in this study experienced a wide range of symptoms such as confusion, an unpleasant unknown perception of illness and bodily sensations with preserved function. The stroke symptoms described were not as clear and concise as the symptoms presented in the stroke campaigns [14, 15] which could lead to subtle symptoms being misinterpreted and thus more likely to cause delay in contact with medical care. However, if there was delayal to seek care was not investigated in the present study. The inconsistency between the experience and the knowledge gained through campaigns [41] might be confusing for both the person who experiences symptoms as well as for the healthcare providers who may not assess the symptoms as stroke [5, 10]. One study has shown that based solely on the symptoms described in public stroke campaigns, strokes can be missed in up to 14% of the cases due to symptoms other than face drooping, arm weakness or slurred speech. The study suggests an update of the acronym in stroke campaigns representing BE-FAST, to add Balance Issues and Eyesight Changes to Face, Arm, Speech and Time [44]. The results from our study suggest that an update could be worth discussing. In this study it emerged that regardless of symptom expression, the patients had never previously experienced symptoms like those of a stroke. The importance of reacting to new symptoms appears from the results of this study to be crucial. It is known that low awareness of stroke symptoms among the public can delay seeking emergency care [45]. In addition, some chose to wait despite suspecting they were experiencing a stroke [31]. However, studies have shown increased recognition of the warning signs of stroke, especially those targeted by the Face, Arm, Speech, Time (FAST) campaigns. Stroke education campaigns have increased the number of patients who contact emergency medical services [43, 46]. But despite FAST campaigns, the knowledge of stroke symptoms remains poor [47]. Repeated information campaigns appear to be essential for maintaining public knowledge [14]. Stroke campaigns should emphasize the importance of seeking medical care at the slightest suspicion of stroke, even if the symptoms are not typical or not perceived as severe. This knowledge is needed among all healthcare professionals to correctly interpret the patient’s descriptions as stroke symptoms.

We investigated how stroke patients describe their stroke symptoms at onset, as one of few recent qualitative studies that focuses on the topic. This study has several strengths given its heterogeneous samples. A purposeful selection has provided variety in experiences and symptoms descriptions among study participants with a wide age range in both sexes. The sample was relatively large for a qualitative study comprising 27 men and women. Nevertheless, it is possible that if more participants had been included, symptoms that do not appear in this study could have been captured. We included patients admitted to both university hospitals and a local hospital to enable the inclusion of different patient groups. As so the results can be used to broaden the understanding of symptoms at first-time stroke onset and the symptom descriptions reported by the patients in this study could potentially be transferred to similar settings. However, future quantitative research is needed in a larger group of patients to further investigate the aspects presented in this study.

A qualitative manifest content analysis derived from the patients’ descriptions and experience of stroke symptoms was utilized. The analysis was carried out by researchers experienced in qualitative method. Quotes from patients’ descriptions were used to each paragraph and throughout the text to ensure that the reader can assess the trustworthiness of the results. However, the study did not include stroke patients unable to express themselves or did not speak the Swedish language, which is a potential limitation. These groups may have other experiences of stroke onset that were not captured in this study. There is also a risk of recall bias since the interviews took place up to a maximum of 4 weeks from symptom onset. In addition, the study does not clarify the type of stroke the patients suffered, knowledge of cause and location could possibly explain the symptoms that were described. Nor have we tried to clarify which symptoms prompt action to seek emergency medical care, since it was outside the scope of this study and requires a different methodological approach. Despite mentioned limitations, our findings could form a basis for further research of how stroke symptoms onset is expressed. Future studies and development of methods to improve how stroke symptom information campaigns should be issued would be of great interest.

## Conclusions

Stroke can be complex with multiple symptoms at stroke onset. The patients described a variety of complex symptom expressions. Symptoms could be subtle or obvious and appear at the same time. The experience was new and never before experienced and, distressed the patients. Many presented with less typical symptoms that could be hard to recognize as stroke and delay medical care. Public stroke campaigns should emphasize the importance of seeking medical care at the slightest suspicion of stroke to receive adequate care in time. Stroke campaigns could be designed to achieve this. More studies are needed to investigate how that information should be designed.

### Electronic supplementary material

Below is the link to the electronic supplementary material.

Supplementary Material 1

Supplementary Material 2

## Data Availability

Data collection and analysis from this study are not publicly available as the participants only provided informed consent for the use of data for the current study. The data that support the findings of this study are available from the corresponding author upon reasonable request.
