# Supplementary material for: Symptoms at stroke onset as described by patients: a qualitative study
Source: BMC Neurol. 2024 May 3;24:150. doi: 10.1186/s12883-024-03658-4 (PMC11067237; doi:10.1186/s12883-024-03658-4)
Supplement: Supplementary file 2 — Supplementary Material 2 [file 12883_2024_3658_MOESM2_ESM.docx]

**Interview guide: Symptoms at stroke onset as described by patients.**

**Introduction:**

Briefly describe what the interview study is about.

State the purpose of the study:

• *To investigate how patients describe their symptoms at the onset of a first-time stroke.*

**Main questions:**

• Can you tell me what it was like when you first had your stroke?

• Can you describe what symptoms you experienced?

• Can you describe those symptoms?

• How did you experience those symptoms?

**Closing questions**

• Is there anything I haven't asked about now that you would like to tell me about?

• How did it feel to be part of this interview?

• What are you going to do after this?

Thank the patient for participation, confirm the value of what the patient has told you and that you value ​​allocated time.
